# Organisational Determinants of Burnout in Psychiatry Inpatient Services

**DOI:** 10.1192/bjo.2026.11635

**Published:** 2026-06-30

**Authors:** Mohamed Jalloh, Samukeliso Fundira, Vasudevan Krishnan

**Affiliations:** 1 St Georges Hospital, Midlands Partnership University NHS Foundation Trust, Stafford, United Kingdom; 2 Royal Stoke University Hospital, University Hospitals North Midlands, Stoke-On-Trent, United Kingdom

## Abstract

**Aims:**

Burnout among psychiatry inpatient staff is often attributed to challenging patient behaviour; however, organisational conditions may play an equally important role. Staffing adequacy and organisational responses following incidents represent potentially modifiable determinants of staff wellbeing. Therefore, we aim to examine associations between perceived staffing adequacy, post-incident support, and burnout among psychiatry inpatient staff.

**Methods:**

A cross-sectional survey was completed by inpatient psychiatry staff (n=115). Burnout was assessed using a validated 7-item composite scale (α;=0.915). Organisational factors were measured using Likert-scale items assessing perceived adequacy of staffing levels to manage risk safely and perceived support from the organisation following incidents. Multivariable linear regression models were constructed adjusting for professional role group, years working in psychiatry, and work pattern. Effect modification by post-incident support was tested using interaction terms. All statistical analyses were performed using IBM SPSS Statistics.

**Results:**

Lower perceived staffing adequacy was strongly associated with higher burnout in univariable analysis (β=−0.41, p < 0.001), explaining 16.9% of variance in burnout scores. This association remained robust after adjustment for staff characteristics (adjusted B=−0.30, 95% CI −0.45 to −0.16; p < 0.001). Higher perceived post-incident support was independently associated with lower burnout (adjusted B=−0.35, 95% CI −0.49 to −0.20; p < 0.001). There was no evidence that post-incident support modified the association between workplace stressors and burnout (interaction p=0.23).

**Conclusion:**

Perceived staffing inadequacy is a major organisational determinant of burnout among psychiatry inpatient staff. While post-incident support is associated with lower burnout overall, structural factors such as staffing levels appear to play a central role. Interventions aimed at improving staff wellbeing should prioritise workforce capacity alongside supportive organisational practices.

